# The Efficacy of *Plantago major* Seed on Liver Enzymes in Nonalcoholic Fatty Liver Disease: A Randomized Double-Blind Clinical Trial

**DOI:** 10.1155/2021/6693887

**Published:** 2021-03-27

**Authors:** Seyedeh Ferdows Jazayeri, Roshanak Ghods, Fataneh Hashem Dabaghian, Asie Shojaii, Seyed Ali Al-Hadi Moravej, Ebrahim Khadem, Seyed Saeed Seyedian

**Affiliations:** ^1^Research Institute for Islamic and Complementary Medicine, Iran University of Medical Sciences, Tehran, Iran; ^2^Department of Persian Medicine, School of Persian Medicine, Iran University of Medical Sciences, Tehran, Iran; ^3^Department of Traditional Pharmacy, School of Persian Medicine, Iran University of Medical Sciences, Tehran, Iran; ^4^Department of Traditional Medicine, School of Traditional Medicine, Tehran University of Medical Sciences, Tehran, Iran; ^5^Department of Internal Medicine, Faculty of Medicine, Ahvaz Jundishapur University of Medical Sciences, Ahvaz, Iran

## Abstract

**Objective:**

This study aims to evaluate the effects of *Plantago major* (*P. major*) seed on liver enzymes and ultrasound patterns in nonalcoholic fatty liver disease (NAFLD).

**Design:**

In this randomized double-blind placebo-controlled clinical trial, 74 patients with NAFLD were administered either 2 g *P. major* or placebo twice daily for 12 weeks. All patients were advised to follow the recommendations for daily exercise and diet modification. Levels of liver enzymes as well as other laboratory indexes were measured at the beginning of the study and after 12 weeks. Indeed, the alteration in ultrasound grade was evaluated in NAFLD patients.

**Results:**

Sixty-three participants completed the study in the intervention and control groups. The mean age of participants was 43.3 (±8.927) and 38.7 (±8.48) years in the intervention and control groups, respectively. *P. major* group showed significant reduction in alanine aminotransferase (ALT) (47.32 ± 21.77 IU/L vs. 50.03 ± 21.19, *P* = 0.021), aspartate aminotransferase (AST) (28.29 ± 10.49 IU/L vs. 32.03 ± 13.30, *P* = 0.004), triglyceride (TG) (200.93 ± 106.741 mg/dl vs. 183.75 ± 73.96, *P* = 0.001), waist circumference (WC) (101.25 ± 9.27 cm vs. 101.18 ± 8.63, *P* = 0.027), and grade of fatty liver in ultrasonography (*P* = 0.038), comparing to the placebo group. There was no significant difference between the two groups regarding serum levels of fasting blood sugar (FBS), high-density lipoprotein (HDL), low-density lipoprotein (LDL), cholesterol, and other outcomes.

**Conclusion:**

*P. major* supplementation with a daily dose of 2 g for 12 weeks improved serum levels of ALT, AST, and TG in patients with NAFLD. Further studies with a larger sample size are recommended.

## 1. Introduction

Nonalcoholic fatty liver disease (NAFLD) is a disease with a wide range from a simple accumulation of fat (triglyceride) in liver cells and simple steatosis to the progression to nonalcoholic steatohepatitis (NASH). About 20% of patients with NAFLD develop liver fibrosis, even cirrhosis and liver failure [1]. The high prevalence and chronic nature of NAFLD affect the patients' quality of life and impose a heavy economic burden on society. The prevalence of nonalcoholic fatty liver disease is increasing in the world. In developed countries, about 25% of the total adult population and about 3–10% of children have this disease, and the prevalence of disease among obese children is about 34% [2]. In Iran, at least 30% of the population suffers from this disease [3]. An unhealthy lifestyle is one of the most important risk factors for NAFLD. Excessive food and calorie intake and lack of enough physical activity often lead to obesity and liver steatosis. Excessive consumption of saturated fats and carbohydrates induces lipogenesis in the liver, inflammation in adipose tissue cells, and insulin resistance in adipose tissue, liver, and skeletal muscle [4, 5]. Besides, an unhealthy lifestyle, which seems to be the most important risk factor for NAFLD, and other factors such as aging, genetics, and intestinal dysbiosis (altered normal intestinal flora) which is thought to be caused by an unhealthy diet may lead to fat accumulation in the liver and the pathogenesis of NASH [6–8]. Besides, the patients with hepatic fibrosis and NASH are also asymptomatic; they may experience fatigue, boredom, right upper quadrant (RUQ) discomfort, or severe symptoms of chronic liver disease [9].

Although liver biopsy is the most specific test to assess the nature and severity of the liver disease, however, liver ultrasound is the easiest diagnostic method for hepatic steatosis [10, 11]. Different clinical trials for testing modern drug candidates of NASH have failed to reach the major findings or have limited therapeutic efficacy. Several agents like nuclear receptor agonists (obeticholic acid, GFT505, and elafibranor), insulin sensitizers (glitazones, pioglitazone, and metformin), and glucagon-like peptide-1 receptor agonists are still in the drug pipeline for NASH [12].

Persian Medicine (PM) has considered a special role for the liver to maintain human health so that the scholars of PM, including Ibn Sina, have considered the liver as one of the chief organs of the body along with the heart and brain [13]. Due to PM, the liver, as the main source of production of humors (phlegm, yellow and black bile, and sanguine) plays an important role in human life and health [14].

Various plants were used to treat liver diseases in PM with different mechanisms. Several herbal remedies show a potential benefit for NAFLD management [15]. Different studies have investigated the efficacy of some of these plants such as turmeric, barberry root, shallot, and rose, on fatty liver [16–19]. *P. major* (Barhang in Persian) is one of the herbs mentioned as a liver tonic in PM literature which can open liver obstructions. *P. major* (plantaginaceae) is a perennial plant that widely grows in the United States, Europe, and Asia and almost throughout Iran [20]. This plant has various pharmacological compounds such as flavonoids, polysaccharides, terpenoids, lipids, iridoid glycosides, and derivatives of caffeic acid which is used to treat various diseases such as constipation, cough, wounds, infection, fever, inflammation, and bleeding [21]. The hepatoprotective effects of *P. major* were confirmed in animal studies [22–24]. Due to our investigations, the efficacy of *P. major* to treat fatty liver has not been studied yet; hence, this clinical trial was designed to evaluate the effects of *P. major* on liver enzymes and ultrasound patterns in NAFLD.

## 2. Materials and Methods

### 2.1. Trial Design

This study was a randomized, double-blind placebo-controlled clinical trial with two parallel groups of NAFLD patients. Patients who had the inclusion criteria were included in the study and randomly allocated into two groups through the “block randomization” method. This method was utilized using 4-way blocks and a random number table. Interventions A and B accurately defined six blocks of four. Using random numbers, we carefully selected and typically wrote blocks. Each individual was then assigned to an A or B group. Patients in the intervention group (A) and who were in the placebo group (B) received two capsules, each containing 500 mg of *P. major* seed or placebo, twice a day, 30 minutes before a meal or 2 hours after the meal. Both groups carefully were followed up for 12 weeks.

This study was approved by the Medical Ethics Committee of Iran University of Medical Sciences (Reference number: IR.IUMS.REC.1398.412) which was registered in the Clinical Trials Registry (ClinicalTrials.gov ID: IRCT20191006044993N1).

### 2.2. Participants

Seventy-four patients with NAFLD referred to Behesht Persian Medicine clinic affiliated to Iran University of Medical Sciences in Tehran, Iran, and other Persian Medicine clinics in Tehran were enrolled. The study was started from December 2019 to August 2020, and the follow-up period was ended in October 2020.

Inclusion criteria were NAFLD patients aged between 12 and 80 years with liver enzymes rising and ultrasound report grades 1 and 2 of fatty liver. Exclusion criteria were patient disliking to enter this study, pregnancy, lactation, anticoagulant drugs consumption, thyroid diseases, spleen diseases, cirrhosis, viral hepatitis and obstructive diseases of liver, uncontrolled diabetes mellitus, being under the treatment of dyslipidemia, lung diseases, malignancy, alcohol consumption, consumption of any drug which affects liver enzymes and liver metabolism like OCP, corticosteroids, salazine, usage of hepatotoxic drugs within the past 6 months, and renal insufficiency (serum creatinine ≥1.5 mg/dl).

Patients included in this study should be visited by a gastroenterologist and had a report of ultrasound confirming NAFLD and ruled out other possible causes of liver diseases (alcoholic fatty liver, Wilson, hypothyroidism, infectious, autoimmune, pharmacological and reactive hepatitis, etc.). Patients and investigators were blind to the intervention and control groups.

### 2.3. Intervention

After signing a written informed consent and taking a complete history of patients at the start of the study, they were randomly divided into two groups of case and control. Patients in both groups were given dietary recommendations due to classical medicine and recommended walking at an average speed for 40 minutes daily. Patients in the treatment group received two capsules (each containing 500 mg *P. major* seed) at 10 a.m. and two capsules at 6 p.m. for a period of 12 weeks. In the control group, patients received placebo capsules (two 500 mg capsules, two times a day) for 12 weeks. *P. major* and placebo capsules were packaged in similar containers and labeled accordingly. Consumption of less than 70% of the drug during the trial was considered as drug intolerance, and the patient was excluded from the trial.

This study's primary outcome was the evaluation of *P. major* on liver enzymes (AST >38, ALT >40) after the 12^th^ week, focusing on ALT changes as the main enzyme showing liver diseases.

Due to similar studies biochemical tests, complete blood count (CBC), FBS, TG, total cholesterol, HDL cholesterol, LDL cholesterol, and specific gravity (SG) of urine were also measured at weeks 0 and 12^th^ [25].

Blood samples and urine analysis were taken from all patients after 12 h of fasting. All participants were evaluated according to blood pressure, height, weight, and waist circumference, at the beginning and the end of the study. The BMI was calculated with the following formula: BMI = kg (weight)/m^2^ (height) [26].

A standard flexible tape was used to measure waist circumferences in standing position, and with a standard scale (Omrone with ±0/5 kg); body weight of patients was measured. To avoid measurement error, all measurements were accomplished by the same person.

Photometric assay (Pars Azmoun Company) for the measurement of ALT and AST and enzymatic (glucose oxidase) colorimetric method using the standard kits (Pars Azmoun Company, Iran) to measure FBS were used. Secondary outcome measures were changes in fatty liver's grade (fatty tissue infiltration in the liver by using ultrasound imaging). The severity grade of fatty infiltration is determined by ultrasound due to the standard structure documented in Goldberg textbook:

  Grade 0 is considered to be the normal liver echogenicity.
  Grade 1 (mild): echogenicity is slightly increased, with normal visualization of the diaphragm and the intrahepatic vessel borders.
  Grade 2 (moderate): echogenicity is moderately increased with slightly impaired visualization of the diaphragm or intrahepatic vessels.
  Grade 3 (severe): echogenicity is markedly increased with poor visualization of the diaphragm, the intrahepatic vessels, and the posterior portion of the right lobe [27].

### 2.4. Drug Preparation

*P. major* seeds were purchased from medicinal plants store in Tehran which were authenticated by a botanist from School of Pharmacy, Tehran University of Medical Sciences, Tehran, Iran (Voucher no. PMP-1748). Each drug capsule was filled with 500 mg *P. major* seed. In parallel, placebo capsules were filled with 500 mg toasted flour powder and were packaged in similar containers and labeled accordingly under a standard condition.

### 2.5. Total Phenolic and Flavonoid Content of *P. major* Seeds

The total phenolic content (TPC) of *P. major* seed was determined using the Folin–Ciocalteu method with some modifications [28].

For the preparation of the calibration curve, 1 ml aliquots of 75, 100, 150, and 200 *μ*g/ml hydroethanolic (50 : 50) gallic acid solutions was mixed with 5 ml Folin–Ciocalteu reagent and 3 ml sodium carbonate (2% w/v). The absorption was read after 2 h at 760 nm and the calibration curve was drawn. One ml of *P. major* seed extract (1 mg/ml) was mixed with the same reagents as described above, and the absorption was measured for the determination of plant phenolic contents. All determinations were performed in triplicate.

The total flavonoid content (TFC) of *P. major* seed was determined using AlCl_3_ reagent. Briefly, 1 ml of each sample (and/or catechin as the standard), previously dissolved in 90% ethanol, was mixed with 0.2 ml of NaNO_2_ 5%. After 5 minutes, we added 0.3 ml of AlCl_3_ solution 3% and 2 ml of NaOH 2M. After 30 min, the absorbance was measured at 510 nm [29].

### 2.6. Sample Size

The final sample size was calculated using a similar study [30], considering a power of 80% and *α* = 0.05. By the expectation of potential loss to follow-up, 74 patients (37 persons in each group) were enrolled.

### 2.7. Statistical Analysis

The data were analyzed by SPSS software (version 17). The Kolmogorov–Smirnov test confirmed the normal distribution of variables. The mean ± standard deviation or number and frequency percentage were used to describe the variables. Comparing qualitative variables among groups was made using the Chi-square or Fisher exact test. Quantitative variables were compared among groups using a *t*-test or Mann–Whitney *U* test. *P* values less than 0.05 (*P* < 0.05) were considered statistically significant.

### 2.8. Safety Assessment

At the beginning of the study, a form was provided to patients in each group for recording possible drug side effects such as gastrointestinal symptoms, constipation, diarrhea, and the effect on libido.

## 3. Results

### 3.1. Total Phenolic and Flavonoid Contents

TPC of *P. major* seed was calculated as mean ± standard deviations of *μ*g of gallic acid (GAE) equivalents/mg extract (*y* = 0.4393*x* + 0.0181, *r*^2^ = 0.9928). TPC of *P. major* seed was 4 ± 0.2 mg/gram (mean ± SE *n* = 3).

TFC (as *μ*g catechin equivalents/mg of a sample) for the sample was calculated on the basis of a linear calibration curve obtained using catechin (*Y* = 0.0169 *x* + 0.3526, *r*^2^ = 0.999). TFC of *P. major* seed was 60 ± 0.22 mg/gram (mean ± SE *n* = 3).

### 3.2. Clinical Trial Results

**Figure 1:**
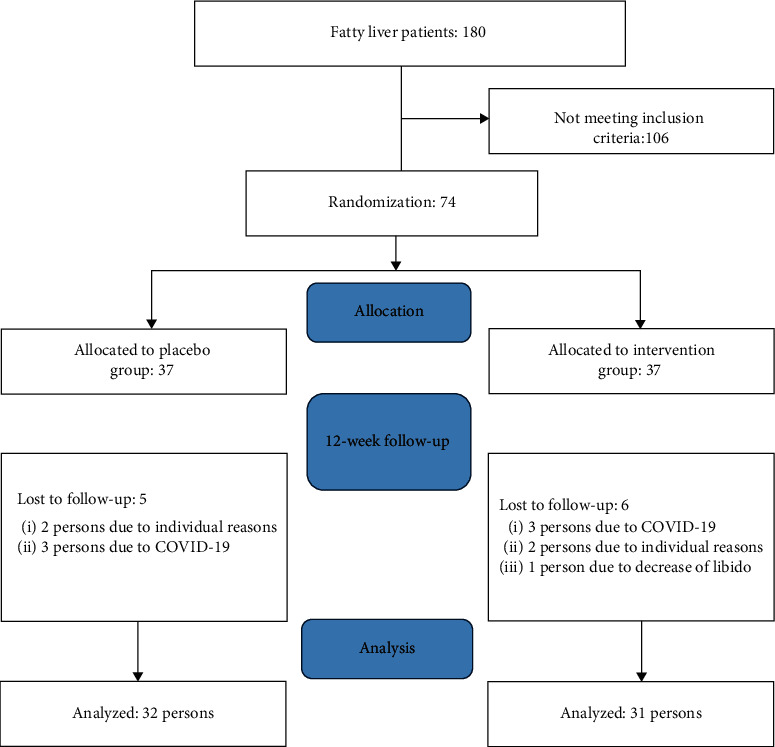
Consort flow diagram of the study.

180 patients were enrolled and 106 subjects who had not met the inclusion criteria were excluded (Figure 1).

Seventy-four patients who met the inclusion criteria and agreed to participate in the study were divided into two groups. Thirty-seven patients were assigned to the intervention group and 37 patients to the control group. Six individuals were excluded from the intervention group for reasons (three patients due to COVID-19, two patients due to personal reasons, and 1 patient due to the decrease of libido) and five individuals left the placebo group (three patients due to COVID-19 and two patients due to personal reasons). Finally, 31 individuals in the intervention group and 32 individuals in the placebo group completed the study which was assessed after 12 weeks.

Baseline characteristics of study groups are summarized in Table 1. Due to demographic characteristics, the mean age of participants was 43.3 (±8.927) and 38.7 (±8.48) years in the intervention and control groups, respectively. There were no significant differences in baseline demographic data and age, gender, and BMI between the two groups (Table 1).

Regarding laboratory indices, there were no significant differences in FBS, grade of NAFLD in ultrasound, ALT and AST, TG, cholesterol, and urine specific gravity levels between the two groups at the beginning of the study (Table 2).

Significant differences were observed due to baseline HDL (*P* value = 0.029), LDL (*P* value = 0.036), and platelet (*P* value = 0.038) (Table 2).

Following the patients in both groups, compliance with lifestyle did not show any significant differences between groups. We followed up the patients in both groups in the terms of compliance with lifestyle modification every week, and reminded them to do the diet and exercise instructions. Weight loss between three to 5% during 12 weeks was considered to assess the compliance with lifestyle and exercise modification between the two groups; at the end of the study, the weight change in the participants of the two groups was about three to 5% (Table 1). The analysis of results after 12 weeks reflected that liver enzymes (ALT and AST) decreased in both groups; however, the percent changes of liver enzymes in the intervention group were significantly higher than the placebo group (ALT, *P* value = 0.021; AST, *P* value = 0.004), and TG decreased significantly in the intervention group (*P* = 0.001) (Table 2).

In both groups, anthropometric factors such as weight, WC, and BMI reduced after 12 weeks; especially WC was decreased significantly in the intervention group (*P* value = 0.027). There was a significant reduction in the grade of NAFLD over the study period in the intervention group compared with the control group (*P* value = 0.038) (Table 2).

There was no report of severe adverse effects in patients of both groups; only one patient in *P. major* group complained of decreasing libido, which was excluded from the study.

## 4. Discussion

This is the first clinical trial investigating the effect of *P. major* supplementation on NAFLD. In this randomized double-blind clinical trial, after 12 weeks, compliance of lifestyle modifications was the same in both groups. There was no significant difference in BMI between the two groups. Due to various studies, it showed that 5–10% weight loss is recommended for the effectiveness of the dietary intake and physical activity at improving NAFLD [31]. The percentage of weight loss was less than 5% in both groups (Table 1).

The mean ALT and AST enzymes, WC, and TG were significantly decreased. Also, *P. major* leads to a significant reduction in ultrasound grade of fatty liver in patients with NAFLD, compared with placebo. In a study by Ramezani et al., *P. major* showed a significant reduction in ALT and AST enzymes in mice compared to the control group, which supported the present study's findings [32]. Waist circumference measurement was used to diagnose and define central obesity. This type of obesity is related to visceral fat, insulin resistance (IR), and increased free fatty acid levels [33]. WC is significantly decreased in both groups; it was more significant in *P. major* group (*P* = 0.027). This reduction in WC may be related to the improvement of gastric digestion [34] and the reduction of gastrointestinal bloating [35]. *P. major* showed efficacy in the regulation of carbohydrate and fatty acids metabolism in an animal study [32]. In another study, some other species of Plantaginaceae, such as *P. lanceolata* showed effectiveness to prevent obesity in mice by stimulating metabolism throughout visceral fat tissue [36].

Due to PM, as well as in classical medicine, the first step to treat fatty liver disease is lifestyle modification. Both groups were assigned similar diet and physical activity due to classical medicine supervised by a gastroenterologist. The placebo group also showed a significant reduction in weight, BMI, and liver enzymes at the end of the study, and this confirms that lifestyle modification is the most important factor in the treatment and improvement of NAFLD indices. Although in the early stages of NAFLD, when the prevalence of NASH and advanced fibrosis is low, 5–8% weight loss and a healthy diet may be sufficient for treatment; in the more advanced stages of liver disease, lifestyle modification with medication may be necessary [2].

Several experimental studies indicated the hepatoprotective effects of *P. major* and confirmed this study's findings. *P. major* showed potent antioxidant activity. Natural products with antioxidant properties showed hepatoprotective activity against some liver toxicity [37]. Turel et al. indicated the protective effects of *P. major* on liver cells in mice poisoned by carbon tetrachloride [22]. Mello et al. showed that *P. major* could prevent oxidative damage to mitochondria, known for its hepatoprotective effect against the toxic effects of oxygen radicals [24]. Scarlat et al. showed the protective effects of *P. major* on liver cells in mice poisoned by diclofenac. *P. major* leads to a decrease in triglycerides values [38]. In a similar study, Nasr et al. indicated that methanolic extract of *P. major* had hepatoprotective effects against hepatocyte damage caused by carbon tetrachloride in mice and reduced the levels of liver enzymes and TG in sick mice. In fact, the antioxidants, as well as anti-inflammatory effects of the flavonoid compounds present in *P. major* reveal its hepatoprotective role. Flavonoids are known as antioxidants, free radical scavengers, and antilipoperoxidants leading to hepatoprotection [21]. The anti-inflammatory activity of *P. major* is through an inhibitory effect on lipoxygenase enzyme. This enzyme catalyzes arachidonic acid to produce leukotrienes. Leukotrienes play a role in inflammatory diseases. The anti-inflammatory activity of *P. major* is exerted by flavonoids such as Baikaline and Hispidoline and iridoid glycosides [39]. These findings are consistent with this study's findings.

There were some limitations like the short duration of the study and the use of *P. major* seed as a capsule for better acceptance in patients (in PM its decoction form is recommended). The effect of *P. major* on liver histology (liver biopsy) was unstudied because it was quite invasive. The prevalence of COVID-19 pandemic prevents patients from participating in the study or causes them to leave the study; also, due to COVID-19 pandemic, the patients (who lived in different parts of the city) preferred to go to the nearest medical center for ultrasound (before and after 12 weeks).

## 5. Conclusions

This randomized, double-blind controlled clinical trial indicated that treatment with *P. major* seed could reduce liver enzymes and improve the grade of fatty liver in NAFLD. Regarding cost-effectiveness and availability of *P. major* and the lack of any reports for serious complications of this plant, *P. major* can be recommended in patients with NAFLD. Further research with larger sample size, as well as long-term follow-ups of patients, can provide a stronger document about the usage of *P. major* as complementary medicine to treat NAFLD.

## Figures and Tables

**Table 1:** Basement characteristics of *P. major* and placebo group.

Variable	Group	Baseline	*P* value	After 12 weeks	*P* value^3^	Percent change	*P* value^3^
Gender (M/F)	*P. major*	M = 28, F = 3	0.113^1^				
	Placebo	M = 32, F = 0					
Age (y)	*P. major*	43.3 ± 8.92	0.054^2^				
	Placebo	38.7 ± 8.48					

WC (cm)	*P. major*	104.58 ± 8.89	0.606^3^	101.25 ± 9.27	0.978	3.2 ± 2.3	0.027
	Placebo	103.65 ± 8.88		101.18 ± 8.63		2.33 ± 2.7	
BMI (kg/m^2^)	*P. major*	29.68 ± 3.19	0.83^3^	28.74 ± 3.15	0.842	3.15 ± 1.8	0.262
	Placebo	30.0 ± 3.85		29.17 ± 3.76		2.73 ± 3.3	

^1^Fisher's exact test; ^2^*t*-test; ^3^Mann–Whitney *U* test. WC = waist circumference; BMI = body mass index.

**Table 2:** Comparing biochemical parameters and NAFLD grades at the baseline and after 12 weeks in *P. major* and placebo group.

Variables	Group	Baseline	*P* value^1^	After 12 weeks	*P* value	Percent change	*P* value^1^
ALT (IU/L)	*P. major*	79.87 ± 36.19	0.068	47.32 ± 21.77	0.474	35.94 ± 22.82	0.042
	Placebo	66.37 ± 25.54		50.03 ± 21.19		19.53 ± 28.72	

AST (IU/L)	*P. major*	43.77 ± 17.24	0.106	28.29 ± 10.49	0.39	29.59 ± 24.31	0.004
	Placebo	37.93 ± 16.80		32.03 ± 13.30		12.09 ± 19.97	

FBS (mg/dl)	*P. major*	99.0 ± 12.02	0.429	103.93 ± 14.41	0.079	−5.48 ± 12.85	0.67
	Placebo	100.43 ± 25.8		103.62 ± 41.95		−2.59 ± 15.23	

Cholesterol (mg/dl)	*P. major*	190.83 ± 42.72	0.559	183.12 ± 42.86	0.726	3.27 ± 15.05	0.157
	Placebo	189.65 ± 35.49		185.68 ± 33.84		1.60 ± 7.65	

TG (mg/dl)	*P. major*	230.03 ± 108.02	0.063	200.93 ± 106.74	0.929	6.81 ± 36.45	0.001
	Placebo	181.31 ± 77.33		183.75 ± 73.96		−5.07 ± 23.97	

HDL (mg/dl)	*P. major*	40.19 ± 7.11	0.029	42.67 ± 6.71	0.05	−7.73 ± 17.04	0.433
	Placebo	36.12 ± 4.03		39.37 ± 5.03		−9.46 ± 12.74	

LDL (mg/dl)	*P. major*	104.90 ± 32.55	0.036	106.41 ± 33.24	0.466	−4.79 ± 33.39	0.326
	Placebo	114.03 ± 26.68		108.62 ± 25.72		4.27 ± 8.56	

Platelet	*P. major*	218.03 ± 63.50	0.038	213.09 ± 64.81	0.018	1.15 ± 14.23	0.773
	Placebo	247.30 ± 73.20		244.28 ± 76.66		−0.84 ± 17.62	

Urine S. G	*P. major*	1020.60 ± 5.19	0.577	1021.40 ± 5.08	0.827	−0.07 ± 0.51	0.607
	Placebo	1021.21 ± 6.90		1021.87 ± 7.80		−0.06 ± 0.44	

Ultrasound grade *n* (%)	*P. major*/placebo	31/32	0.572^2^	31/32			
	Grade 0	0/0		3 (9.6)/0	0.038^2^
	Grade 1	9 (29.1)/7 (21.9)		17 (54.8)/8 (25)	
	Grade 2	22 (70.9)/25 (78.1)		11 (35.5)/23 (71.9)	
	Grade 3	0/0		0/1 (3.1)	

^1^Mann–Whitney *U* test; ^2^Pearson chi-square. ALT, alanine aminotransferase; AST, aspartate aminotransferase; FBS, fasting blood sugar; CHOL, cholesterol; TG, triglyceride; HDL, high-density lipoprotein; LDL, low-density lipoprotein; SG, specific gravity of urine; negative sign, increase.

## Data Availability

The data used to support the findings of this study are included within the article.

## References

[1] Kohnagi K., Asadollahi K., Abangah G., Miri S. (2016). Evaluation of risk factors for nonalcoholic fatty liver disease: a case-control study. *Tehran University of Medical Sciences TUMS*.

[2] Stefan N., Häring H.-U., Cusi K. (2019). Non-alcoholic fatty liver disease: causes, diagnosis, cardiometabolic consequences, and treatment strategies. *The Lancet Diabetes & Endocrinology*.

[3] Moghaddasifar I., Lankarani K. B, Moosazadeh M (2016). Prevalence of non-alcoholic fatty liver disease and its related factors in Iran. *International Journal of Organ Transplantation Medicine*.

[4] Donnelly K. L., Smith C. I., Schwarzenberg S. J., Jessurun J., Boldt M. D., Parks E. J. (2005). Sources of fatty acids stored in liver and secreted via lipoproteins in patients with nonalcoholic fatty liver disease. *Journal of Clinical Investigation*.

[5] Stefan N., Kantartzis K., Häring H.-U. (2008). Causes and metabolic consequences of fatty liver. *Endocrine Reviews*.

[6] Angulo P., Kleiner D. E., Dam-Larsen S. (2015). Liver fibrosis, but no other histologic features, is associated with long-term outcomes of patients with nonalcoholic fatty liver disease. *Gastroenterology*.

[7] Stefan N., Schick F., Häring H.-U. (2017). Causes, characteristics, and consequences of metabolically unhealthy normal weight in humans. *Cell Metabolism*.

[8] Marra F., Svegliati-Baroni G. (2018). Lipotoxicity and the gut-liver axis in NASH pathogenesis. *Journal of Hepatology*.

[9] Preiss D., Sattar N. (2008). Non-alcoholic fatty liver disease: an overview of prevalence, diagnosis, pathogenesis and treatment considerations. *Clinical Science*.

[10] Tapper E. B., Lok A. S.-F. (2017). Use of liver imaging and biopsy in clinical practice. *New England Journal of Medicine*.

[11] Hernaez R., Lazo M., Bonekamp S. (2011). Diagnostic accuracy and reliability of ultrasonography for the detection of fatty liver: a meta-analysis. *Hepatology*.

[12] Shi T., Wu L., Ma W. (2020). Nonalcoholic fatty liver disease: pathogenesis and treatment in traditional Chinese medicine and western medicine. *Evidence-Based Complementary and Alternative Medicine*.

[13] Toosi M. N., Ardekani M. R. S., Esfahani M. M., Bagher Minaei M., Nazim I., Khadem E. (2016). Fatty liver disease from the perspective of traditional Iranian medicine. *Quran Medicine*.

[14] AAH I. (1866). *Al-Qanun Fi Al-Tibb*.

[15] Hosseini S. M.-R., Razmgah G. R. G., Nematy M. (2018). Efficacy of black seed (Nigella sativa) and lemon balm (melissa officinalis) on non-alcoholic fatty liver disease: a randomized controlled clinical trial. *Iranian Red Crescent Medical Journal*.

[16] Davoodi I., Rahimi R., Abdollahi M. (2017). Promising effect of Rosa damascena extract on high-fat diet-induced nonalcoholic fatty liver. *Journal of Traditional and Complementary Medicine*.

[17] Kazemi S., Asgary S., Moshtaghian J., Rafieian M., Adelnia A., Shamsi F. (2010). Liver-protective effects of hydroalcoholic extract of allium hirtifolium boiss. in rats with alloxan-induced diabetes mellitus. *Arya Atherosclerosis*.

[18] Rahmani S., Asgary S., Askari G. (2016). Treatment of non-alcoholic fatty liver disease with curcumin: a randomized placebo-controlled trial. *Phytotherapy Research*.

[19] Taheri S., Zarei A., Ashtiyani S. C., Rezaei A., Zaheiri S. (2012). Evaluation of the effects of hydroalcoholic extract of Berberis vulgaris root on the activity of liver enzymes in male hypercholesterolemic rats. *Avicenna Journal of Phytomedicine*.

[20] Samuelsen A. B. (2000). The traditional uses, chemical constituents and biological activities of *Plantago major L*. a review. *Journal of Ethnopharmacology*.

[21] Najafian Y., Hamedi S. S., Kaboli Farshchi M., Feyzabadi Z. (2018). Plantago major in traditional persian medicine and modern phytotherapy: a narrative review. *Electronic Physician*.

[22] Türel I., Özbek H., Erten R., Öner A. C., Cengiz N., Yilmaz O. (2009). Hepatoprotective and anti-inflammatory activities of Plantago major L. *Indian Journal of Pharmacology*.

[23] Nasr S. M., Mouneir S. M. (2006). Potential protective effect of some plant extracts against carbon tetrachloride–induced hepatotoxicity. *African Journal of Traditional, Complementary and Alternative Medicines*.

[24] Mello J. C., Guimarães N. S. S., Gonzalez M. V. D. (2012). Hydroxyl scavenging activity accounts for differential antioxidant protection of *Plantago major* against oxidative toxicity in isolated rat liver mitochondria. *Journal of Pharmacy and Pharmacology*.

[25] Panahi Y., Kianpour P., Mohtashami R., Jafari R., Simental-Mendía L., Sahebkar A. (2017). Efficacy and safety of phytosomal curcumin in non-alcoholic fatty liver disease: a randomized controlled trial. *Drug Research*.

[26] Misra A., Dhurandhar N. V. (2019). *Current Formula for Calculating Body Mass Index Is Applicable to Asian Populations*.

[27] Scatarige J. C., Scott W. W., Donovan P. J., Siegelman S. S., Sanders R. C. (1984). Fatty infiltration of the liver: ultrasonographic and computed tomographic correlation. *Journal of Ultrasound in Medicine*.

[28] Haile M., Kang W. (2019). Antioxidant activity, total polyphenol, flavonoid and tannin contents of fermented green coffee beans with selected yeasts. *Fermentation*.

[29] Greenberg A. D., Connors J. J., Jenkins D. M. A. (1981). *Franson Standard Methods for the Examination of Water and Wastewater*.

[30] Zamani N., Shams M., Nimrouzi M. (2018). The effects of Zataria multiflora Boiss. (Shirazi thyme) on nonalcoholic fatty liver disease and insulin resistance: a randomized double-blind placebo-controlled clinical trial. *Complementary Therapies in Medicine*.

[31] Kenneally S., Sier J. H., Moore J. B. (2017). Efficacy of dietary and physical activity intervention in non-alcoholic fatty liver disease: a systematic review. *BMJ Open Gastroenterology*.

[32] Ramezani M., Changizi-Ashtiyani S., Sadeghzadeh F., Hosseini S.-S., Zarei A., Hosseini N. (2018). Effect of hydroalcoholic extract of some medicinal plants on obesity. *European Journal of Medicinal Plants*.

[33] Rocha R., Cotrim H. P., Carvalho F. M., Siqueira A. C., Braga H., Freitas L. A. (2005). Body mass index and waist circumference in non-alcoholic fatty liver disease. *Journal of Human Nutrition and Dietetics*.

[34] Aghili M. (2008). *Makhzan-O-L Advieh.*.

[35] Agah S. H., Fatali S., Ashayeri N. (2010). Treatment of diarrhea predominant irritable bowel syndrome (IBS-D) using plantagel: a random double-blind clinical trial. *Razi Journal of Medical Sciences*.

[36] Yoshida T., Rikimaru K., Sakai M., Nishibe S., Fujikawa T., Tamura Y. (2013). Plantago lanceolataL. leaves prevent obesity in C57BL/6 J mice fed a high-fat diet. *Natural Product Research*.

[37] Hussan F., Osman Basah R. H., Mohd Yusof M. R., Kamaruddin N. A., Othman F. (2015). Plantago major treatment enhanced innate antioxidant activity in experimental acetaminophen toxicity. *Asian Pacific Journal of Tropical Biomedicine*.

[38] Scarlat M. E., Bucă M., Cristina Zagardan M. (2017). Study on the hepatoprotective effect of plantago major extract on experimental poisoning by diclofenac in NMRI albino mice. *Current Trends in Natural Sciences*.

[39] Hussan F., Mansor A. S., Hassan S. N. (2015). Anti-inflammatory property of *Plantago major* leaf extract reduces the inflammatory reaction in experimental acetaminophen-induced liver injury. *Evidence-Based Complementary and Alternative Medicine*.

